# Voices to be heard: Understanding family perspectives in forensic care trajectories

**DOI:** 10.3389/fpsyt.2022.1022490

**Published:** 2022-12-15

**Authors:** Sara Rowaert, Marjolein De Pau, Florian De Meyer, Pablo Nicaise, Freya Vander Laenen, Wouter Vanderplasschen

**Affiliations:** ^1^Department of Special Needs Education, Ghent University, Ghent, Belgium; ^2^Department of Criminology, Criminal Law and Social Law, Institute for International Research on Criminal Policy (IRCP), Ghent University, Ghent, Belgium; ^3^Faculty of Public Health, Institute of Health and Society (IRSS), Catholic University of Louvain, Brussels, Belgium

**Keywords:** forensic psychiatry, Persons labeled as Not Criminally Responsible, family recovery, care trajectories, qualitative research

## Abstract

**Introduction:**

De-institutionalization of psychiatric care has greatly increased the role of family members in the recovery pathways of Persons labeled as Not Criminally Responsible (PNCR). However, the role of family members in supporting PNCR in forensic psychiatric care remains understudied. Scarce evidence indicates that PNCR have to deal with stigma and endure specific burdens (i.e., symptom-specific, financial, social, and emotional). Recovery-focused research showed that recovery in both persons with a severe mental illness and family members develop in parallel with each other and are characterized by similar helpful principles (e.g., hope and coping skills). As such, the recovery pathways of PNCR often goes hand in hand with the recovery pathway of their family members. During the family recovery process, family members often experience not being listened to or being empowered by professionals or not being involved in the decision-making process in the care trajectory of their relative. Therefore, the aim of this study is to capture how family members experience the care trajectories of their relatives, more specifically by looking at family recovery aspects and personal advocacy of family members.

**Methods:**

Semi-structured interviews were conducted with 21 family members of PNCR from 14 families. A thematic analysis confirms that family members suffer from stigma and worry significantly about the future of their relative.

**Results:**

Regarding the care trajectory of PNCR, family members experienced barriers in multiple domains while trying to support their relative: involvement in care and information sharing, visiting procedures, transitions between wards, and the psychiatric and judicial reporting by professionals. In addition, family members emphasized the importance of (social) support for themselves during the forensic psychiatric care trajectories and of a shared partnership.

**Discussion:**

These findings tie in with procedural justice theory as a precondition for family support and family recovery within forensic psychiatric care.

## Introduction

Persons labeled as Not Criminally Responsible (PNCR) are people considered not responsible for their offenses due to their mental illness. They are diverted toward secure or forensic mental health services, stratified according to different therapeutic security levels (i.e., high, medium or low) (1). A forensic care trajectory intends to promote recovery and desistance from crime and allows PNCR to progress through different security level services according to their security needs, which ultimately leads to the abrogation of the security measure (2–4). Throughout the last decades, de-hospitalization and community-oriented mental healthcare have become central concepts in (forensic) mental healthcare, which challenge the more traditional service-oriented residential care (5). For instance, the Belgian Federal policy plan of 2016–2020 of the former Public Health and Justice Ministers specifically stated that the reintegration of PNCR into society is one of its main goals. As such, the focus has shifted from “illness” to a first person-centered perspective that emphasize the importance of the social resources of the user. This paradigm shift has increased the role and importance of informal carers, more specifically family members, in the (forensic) service users' care trajectory (6–8).

In general, caring for a PNCR can place a burden on family members [e.g., (9, 10)], mainly on mothers, who feel restricted by professionals in their caring role, while at the same time being regarded as a major resource for their child, specifically in the social (re)integration process (6, 8). Family members explain that taking on a primary care role (where they are often forced into by professionals), challenges and burdens them (i.e., socially, financially and emotionally), because they lack knowledge, skills or professional guidance and support to cope with the situation of their relative (8, 11–13). In addition, family members of PNCR are confronted with a double stigma as their relative is not only seen as a “patient” but also as a “criminal” (14, 15). Because of this, family members find it difficult to talk to others (i.e., friends or family members), because they anticipate a lack of full understanding of the situation. They also believe that talking about the situation to others would upset their relative. Therefore, it is possible for family members to become socially isolated (16–18). Nonetheless, throughout different studies, hope is regarded as an important source of strength for family members and essential to motivate their caregiving (7, 9).

Dewaele et al. (19) and Kim and Salyers (20) state that the (re)integration of persons with severe mental illness in society should be a shared responsibility between primary caretakers, professionals and different societal actors to reach inclusive citizenship of these persons. Within forensic mental healthcare, rehabilitation and (re)integration are regarded as complex goals, which challenges the personal recovery process of PNCR, with “time” playing a key role (e.g., having to deal with restrictions and an undefined duration of the security measure) (21). Therefore, it is claimed that empowering family members in their caring role is of great importance for the recovery process and thus (re)integration of PNCR, with professionals and therapists being considered as guides and coaches (22). Still, including family members as partners in care continues to be a challenge in forensic mental healthcare (23, 24). Family inclusion or family interventions in treatment settings are often found to be limited from a family member perspective [e.g., (7, 8, 11)]. For instance, Canning et al. [(25), p. 877] mention that support for carers “*has not yet become a consistent or widespread part of forensic services practice*.” Family members of PNCR tend to describe the relationship with professionals as a “terrible battle,” because they feel not listened to and experience no effective partnership (7, 16, 26). Furthermore, a lack of information often leads to family members feeling uninformed, isolated and detached (7, 9). Nonetheless, taking on a caring role and caring responsibilities influences the family members' self-care and autonomy (7, 18), making it clear that family members are going through their own recovery process and have to deal with the consequences of the mental illness, the criminal offense and recovery process of their relative (24).

In recent years, recovery research and recovery-based practice paid more attention to the importance of living a qualitative, satisfying, empowering and hopeful life for PNCR (21, 27, 28). However, research indicates how the organization of care (trajectories) might also be a barrier to recovery of PNCR. For example, they often feel they do not have a voice in the decisions made concerning their trajectory, which is contradictory to the empowerment principle of recovery. In addition, due to in- and exclusion criteria in forensic services PNCR are regularly deprived of the (specialized) care they need (29, 30). Perspectives of family members are only rarely addressed within forensic recovery literature (28). In this respect, reflecting upon whether family members have similar experiences (i.e., lack of voice and support) and how the organization of care (trajectories) hamper family recovery, can be of interest for both the PNCR's recovery process, as well as for the professionals supporting both PNCR and their family.

Non-forensic empirical studies found that recovery in both persons with a severe mental illness and family members develop in parallel with each other and are characterized by similar principles (e.g., hope and coping skills) (31–34). Family recovery consists of individual and relational processes (33), with family members going through their own recovery process, which can ultimately enhance their quality of life and help them to support the recovery process of their relative (31). Family recovery consists of a series of phases: (1) shock, discovery and denial, (2) recognition and acceptance, (3) coping and (4) personal and political advocacy (35), where family members move through at different paces (33). Notably, during the family recovery process, many family members keep on being supportive of their relative. Yet, not being listened to by professionals or not being involved in the decision-making process in the care trajectory of their relative, makes it difficult for them to advocate for their family member (7, 12). Therefore, in this study, we aim to capture how family members experience the care trajectories of their relatives, more specifically we look at family recovery aspects and personal advocacy of family members. The objective of the research is: (1) to understand how family members currently experience their involvement in caring for their relative labeled as NCR; (2) to understand what role they envision for themselves in caring for their relative labeled as NCR; (3) to understand how family members see their involvement in the social reintegration of their relative labeled as NCR and (4) to understand how these experiences and reflections are related to family recovery.

## Methodology

### Research setting

In Belgium, PNCR are subjected to a security measure of undefined duration. This measure is intended to protect society and meanwhile providing care to persons with a severe mental illness who cannot be held responsible for their criminal behavior (36, 37). How the security measure will proceed (e.g., admission to a forensic care facility or release on probation) for the PNCR is decided by the Chamber of the Protection of Society (chaired by a judge). This Chamber can also extend or terminate the security measure. In recent decades, the state of Belgium has been condemned several times by the European Court of Human Rights for the inhumane treatment of PNCR in prison facilities (38). As a consequence, the Belgian authorities have developed and significantly increased forensic mental healthcare capacity in designated (forensic) in- and outpatient wards or facilities (39). These (forensic) wards and facilities can be stratified according to the Trinitarian model of therapeutic security in high-, medium- and low-secure wards and facilities (1). The 2014 law on PNCR subscribes that taking care of the PNCR is crucial besides the protection of society. Therefore, in the last decade, family members and how they could be involved came more and more on the policy agenda. This led to the development of a multidisciplinary guideline for mental healthcare professionals in 2020 (i.e., the family reflex) for involving the social context of a patient in mental healthcare. It focuses on four pillars, namely treating, informing, supporting and involving family members in mental healthcare (40).

### Recruitment

This study follows a similar study design as that of De Pau et al. (29) to maximize the comparability of the findings. The target population in this study are family members of PNCR who are currently residing in a high-, medium- or low-secure setting [cf. (29)]. Throughout the study, three different recruitment strategies were used because it is difficult to find family members willing to talk about the situation they are confronted with due to stigma (9). For the first recruitment strategy, all 23 participants in the study of De Pau et al. (29) were asked if they were willing to give an information letter to their family members. Based on this information, family members could contact the researcher if they wanted to participate. Using this strategy, it was however realistic to expect that some of the participants would refuse to contact their family or that some of these family members would refuse to participate. A second recruitment strategy consisted of informing (forensic) mental healthcare services about the study and asking them to distribute a leaflet to family members of PNCR. In this strategy the forensic care facilities in Flanders [high (*n* = 6), and medium care (*n* = 3)] where PNCR are living, were contacted. The last recruitment strategy consisted of contacting non-profit organizations that support family members of PNCR (*n* = 2), for them to distribute an information leaflet about the current research. Family members could then voluntarily contact the research team and consent to participate in the research. We used this strategy to reach PNCR in low secure care, who are often living independently or at home, together with their family.

Inclusion criteria for participation were: (1) a relative of the participant is labeled as Not Criminally Responsible at the time of the interview and (2) the participant is of adult age (18+). At first, family members of PNCR residing in prison were excluded to be in line with the inclusion criteria used in the study of De Pau et al. (29). However, as it was difficult to find family members, the exclusion criteria were changed to exclude family members of PNCR who have never been in a (forensic) mental healthcare setting before.

The study was approved by the Ethics Committee of Ghent University Hospital with Belgian Registration number: B0201836215. All participants provided informed consent for the pseudonymized publication of their interview responses and were informed about their right to withdraw at any time. Next, information was given to them about psychological support they could go to if the interview raised certain difficulties and about activities organized by a family organization in Flanders.

### Sample

Between June and September 2018 interviews were conducted with family members of mentally ill offenders. In total 21 family members of 14 different families^1^ participated in the research of which 13 were female and 8 were male. Making use of the different strategies above, only the second and third ones yielded response from participants. The mean age of the participants was 61 years (range: 47–70). The sample consisted primarily of mothers (*n* = 9) and fathers (*n* = 5), but also a niece, a nephew, an uncle, an aunt, a partner, a sister and a stepfather were interviewed. All family members involved in this study are the main carers or the first point of contact for the PNCR.

At the time of the interview, the participants' relatives were staying in different forensic settings (i.e., high-, medium-, or low-secure). The duration of the security measure ranged from 23 years to 3 months, with an average duration of 7 years. The most common diagnoses of their relative in the sample were psychotic disorders (*n* = 9). The offense(s)of their relative that lead to the security measure was/were vandalism, assault and battery, theft, sexual offense and arson.

An important insight that we want to share is that the participants in this study are all family members that are still maintaining some sort of contact with their relative despite past events or incidents. They have learned, throughout the years, to cope with the situation their relative is in. The family members in this study were able to talk about the impact of the psychiatric diagnosis and about the offense on their lives. In that way the participants differ from other family members that have no or a troubled relationship with their relative or that find the threshold to participate in a research study to high, because of the stigma.

### Data collection

A topic list was used to collect the data and based upon the questions asked in the study of De Pau et al. (29)^2^. The semi-structured interview started with a focus on family recovery: (1) how do family members cope with the security measure of their relative (e.g., How did the security measure impact your personal life?) and (2) how do they cope with the different labels (i.e., psychiatric patient and criminal) of their relative (e.g., In which way has stigma had an influence on the care and support that is being given by mental healthcare facilities and society in general?). Afterwards, the focus shifted to topics linked to personal advocacy: (3) how they are involved in the care trajectory of their relative (e.g., What support and information do you receive in the care trajectory of your relative?) and (4) what their perspective on the future is and how they see the care trajectory of their relative evolve. While discussing these topics, the participants and the interviewer made up a timeline about the care trajectories of the family members' relative, based on the timeline used in the study of De Pau et al. (29). Every admission to a forensic care facility was being reported by mentioning the period of the stay, the place and the security level (high, medium or low). To enhance the focus on the care trajectories, questions were asked about their experiences with the mental healthcare given to their relative, contact with professionals and the transitions between different care facilities.

Interviews were conducted at a location of choice for the participant, which was mostly at their own home. The interviews were conducted in Dutch, because all participants were native speakers. The interviews were audio recorded and had a mean duration of 01:33:58. The range of the interviews starts from 01:07:00 to 02:27:07. All interviews were coded for each family or participant. After verbatim transcription of the interviews, the audiotapes were deleted.

### Data analysis

All interviews were transcribed verbatim and were analyzed with the thematic analysis method. Braun and Clarke [(41), p. 6] describe thematic analysis as*:* “*a method for identifying, analyzing, and reporting patterns (themes) within data. It minimally organizes and describes your data set in (rich) detail*.” By making use of this analyzing method, you can “give voice” to the participants as a researcher. Since this study aims to understand family members' experiences, we opted for a data-driven inductive approach (42). The first author of this paper started by reading the manuscripts of the interview, which were analyzed inductively. This resulted in a coding structure on paper. The codes and themes mentioned, were thoroughly discussed with the second author of this paper, who had done a similar research study with PNCR (29). More specifically, we selected four manuscripts and looked at it, line-by-line, by making use of the coding structure. This process resulted in an adapted coding structure focusing on three themes: (1) experiences with the security measure, (2) experiences of family members with mental healthcare trajectory and (3) the impact of the situation on their own lives. In the next phase, the timelines that were made during the interviews were interpreted by the first author (e.g., how long did a person stay in a forensic care facility, what was the trajectory of the PNCR during the past years, …) to add more information on the care trajectories in the coding structure (e.g., many persons were staying in minimum three different forensic care facilities over the past 2 years, meaning that they have been moving a lot from one place to another, sometimes with a time-out in prison as well). This interpretation of the care trajectories was looked at together with the second author and in relation to the existing coding structure. This led to a final tree structure containing the following themes: (1) experiences with the security measure, (2) causes and consequences of “decisions” made in care trajectories, (3) family recovery processes, and (4) contemplating the future. These themes are further outlined in the results section, where the different subthemes are mentioned for themes two and three. The analysis process was conducted in Dutch, the native language of the authors and the participants in the study. The quotes mentioned in this paper were translated by the authors from Dutch to English.

## Results

### Experiences with the security measure of a relative

At the beginning of the security measure of their relative, many family members did not apprehend its significance, because they lacked knowledge of this type of measure. Some family members were even wrongfully informed by searching for answers on the internet or by contacting professionals (e.g., that the security measure would only last for 3 years). When considering the duration of the security measure, they often equate it to a lifelong sentence. For example, they compare this security measure with the sentencing of convicted persons and believe that PNCR are more severely punished because of the undefined and long duration of the measure. As such, many family members experience a sense of injustice, especially because they were not aware that the security measure could be continued indefinitely.

“*The security measure is so horrible, you have nothing to live for. In fact, it is a lifelong sentence that he gets. […] You can only hope that it will become better*.” (partner, 57 years)

On a positive note, some of the family members also experience the security measure as something that opened their relative's eyes about both their diagnosis and the consequences of their actions. Not being sentenced but treated as a person with a severe mental illness and getting care for psychiatric problem(s), is experienced as positive by the family members. Nevertheless, they are convinced that the care trajectory (i.e., the different stages they have to go through) should be unique for every PNCR. Family members believe that there should be more space for “customized care” and mental healthcare professionals should have more attention to the specific needs and situation of every person individually.

“*There is probably a fixed trajectory they have to follow and that is the same for everyone. But I am of the opinion that this should be more specific, according to the needs and the situation of the person that is staying there*.” (mother, 47 years)

### Causes and consequences of “decisions” made in care trajectories

As mentioned before, family members have diverse opinions about the security measure and the care their relative is receiving. During the interviews, family members talked about different aspects of the care trajectories, which are thematised below.

#### Involvement and information

Many participants want to be readily involved in the care trajectories of their relatives. Yet, this is often not the case, mainly because of reasons of medical confidentiality.

“…* apparently my son did not want to go in therapy there as a result of his psychosis or not, I do not know. You get no information at all about that, no information at all! I only received some information from one mental healthcare professional that sometimes overstepped the bounds of his duty. He also told me: ‘Sir, do not say anything about this to anyone, because I can lose my job if anyone knows.' Of course I did not. I was really happy to get at least a little information*.” (father, 59 years)

The participants in this study are convinced it would be beneficial for both treatment facilities and themselves if they were regularly invited to a consultation with the concerned mental healthcare professionals.

“*They call you and ask you to come, you do it for your child. For them it is also a way to get to know more about the home situation of their patient. It would be nice to be invited and heard as family on a regularly basis*.” (mother, 61 years)

Most participants feel that they are the ones who need to initiate an information exchange or a consultation, and that it is not initiated by the professionals. Everything they know about the daily life of their relative and how he or she is progressing generally comes from the relative him/herself. Therefore, family members state that mental healthcare professionals should take initiative to inform them about the treatment (stages) of their relatives or initiate a consultation throughout the care trajectory.

“*It was extremely difficult to have contact with the healthcare team. There are very negative prejudices against family members. So you really don't feel listened to. There is a lot of suspicion. While we are the closest people (family member or relatives) who can bring something to support the PNCR because we know the person well*.” (father, 66 years)

Most family members search for information themselves, because they usually do not receive it from mental healthcare professionals. In general, family members want to know about the pharmacological treatment of their relative and its effects. Furthermore, they wish for psycho-education about psychiatric problems. Information about the mental healthcare facility (e.g., the do's and don'ts, activities that are being organized for family members) and how the relative progresses in treatment, would also be important to share with family members.

“*Our aunt did not get much information about her son. […] When he was in a forensic psychiatric hospital they did not tell her anything*.” (niece, 61 years)

In general, many family members experienced difficulties in the past in situations where they were not involved. Some participants mention that they are often not contacted when their relative was transferred to another facility or was on probation leave and things “went wrong” (e.g., not going back to the facility). Family members do not primarily mention or talk about their own feeling of safety when a crisis happens. They mention especially the—in their eyes—missed opportunity to be not consulted, because they can offer information about how their relative can be helped when he/she is distressed. Therefore, family members ask to be heard by mental healthcare professionals and to be involved in important decisions made in the care trajectory of their relative.

“*My son can tell something really trustworthy, so the Chamber thought he could go to a setting with open doors. And then within 14 days, it went wrong. I could have said that! […] But then you are not involved in the decision, and that was the basis were it went wrong. We saw it failing and we knew before*.” (father, 59 years)

Yet, there are also a few family members that are generally positive about the care their relative receives and the communication with mental healthcare professionals, as illustrated by this citation.

“*We are invited to come to several activities and we are always asked to come to the parties they organize. […] When I want it, I can always meet with the mental healthcare professionals*.” (mother, 62 years)

#### Transitions between (care) facilities

When considering transitions between different care facilities or from a prison to a care facility, family members are frustrated about the lack of communication between these facilities and the fact that they, as family, are (often) not informed about this transition. Transitions are considered by family members as unhelpful to the recovery of their relative. This goes together with having to build relationships with new people, i.e., other residents and mental healthcare professionals, and telling their stories all over again. In addition, most services seem to work autonomously from each other, with the result of family members experiencing care as rather fragmented, i.e., a lack of consistency in the care trajectory. Moreover, family members would also recommend better linkage between the care episodes in institutions and probation leave, in order to better coordinate care given in the facility and how to respond to their relative when he/she is at home. Family members need to communicate about what happens at home, but feel reluctant to be transparent to professionals, especially as they have the feeling that what they tell would have a negative impact on the care given to their relative in the forensic care facility.

“*There is a need for a connection into the family when he comes back home. Rather than being in the office all the time. And let someone come home and see how it goes. It would be more efficient and unblock situations. While here we are partitioning. There must have more consistency*.” (mother, 61 years)

During the interview, it became clear that many family members feel relieved and satisfied in the case of a transfer from prison to a care facility due to the long-awaited access to care. Nonetheless, the in- and exclusion criteria of care settings are experienced as neither clear nor transparent. Some family members label the non-acceptance of their relative within general mental healthcare facilities as discrimination. Others feel the “care contract” and reasons for exclusion are rather rigid, which seems to give all the responsibility for (the continuity of) care to the parent.

“*And so we make a contract to the patient saying ‘that you cannot do, that you cannot do'… and if you break one of the clauses of the contract, you're outside, that's it. […] [Imitating an old conversation] I (the father) do not ask you (the psychiatrist) to keep my son, if your institution does not agree, but you will find me another institution. We must ensure the continuity of care. I am not a caregiver. I cannot handle it. I can contribute but it is your mission to ensure continuity of care. Otherwise, there is no assistance to anyone in danger. So on, he found another place*.” (father, 66 years)

On those occasions when their relative is recently transferred to a new facility, many family members are invited to a consultation with a psychiatrist, a psychologist or other mental healthcare professionals with or without the presence of their relative. These consultations have the intention to give family members some general information about the facility their relative resides. This initiative is experienced as positive because they feel heard. Still, they often leave with the feeling that they did not get the information they needed (see above). In this respect, some families mention the need for a case manager to support them along the care pathway.

“*It is important for the continuity of care that there can be some kind of person, like a central figure, who can inform you as a family and who can guide you through the care trajectory. Someone who says, he is going to be transferred to that ward on that date. A person that will go with you to the first meeting on the new ward with the mental healthcare professionals working there. Doing that, would ensure that we do not have to start our story from scratch every time*.” (mother, 61 years)

#### Professional reports

The participants in this study also criticized the psychiatric and judicial professional reports about their relative. They believe these reports are generally made by professionals who are not very familiar with their relative. Therefore, they wonder why criminologists, psychiatrists or psychologists take decisions based on, in their perception, general impressions rather than involving other mental healthcare professionals who know their relative through daily contact.

“*His personal mental healthcare worker knows him, but those who do all the testing do not know him. So that are basically people who are unknowing about who he is as a person that are writing something about him in a report*.” (mother, 47 years)

#### Visiting procedure

The participants in this study who visit their relative in a care facility have similar opinions about the visiting procedure(s). For example, they would love to see the room of their relative, yet this seems impossible because of security issues. There are often many procedural rules for what can be brought into the facility or what can be done during the visiting hours, which makes it difficult for family members to prepare for visits. In addition, family members sometimes feel uneasy during the visit, as they experience little privacy, because of the presence of other residents and/or professionals.

“*The first time that I visited him in the forensic psychiatric hospital and I saw him in his own clothes that was… I saw my son back! Going outside the care setting with him, that are experiences… that is very difficult. Because actually those are normal things, but for me, it is not that obvious anymore*.” (mother, 47 years)

### Family recovery processes

#### Impact of the situation on family members' lives

During the interviews family members describe the impact of the situation (i.e., their relative being labeled as Not Criminally Responsible and subjected to a security measure) on their own lives. They feel they have “changed” as a person and would describe themselves as being rather unhappy. In addition, they experience diverse emotional and relational burdens because of the security measure of their relative. For example, some participants went through a marital crisis or eventually broke up with their partners. Other participants emphasized they were lucky to have a strong and loving family to rely on, which helped to cope with the situation.

“*It was not working anymore, also at home. Several times we had a relational crisis, which was leading to a divorce*.” (mother, 47 years)

Many family members experience a range of emotions, such as anger, sadness, frustration, guilt or shame. They perceived that their life has been ruined and destroyed by “others” (e.g., lawyers, mental healthcare professionals, justice). Two participants, mothers of a forensic client, mention they conducted self-harming behavior throughout the years and even had suicidal thoughts as a consequence. These women have been committed to a psychiatric hospital for several days. Parents in particular often feel to have failed in raising their child; they wondered how they did not see what was happening and whether it could all have been avoided.

“*I had the idea: a child should be the better version of myself. So it is a failure, really a failure for myself. I failed in raising my child*.

***Do you have that feeling?***

*I feel like I failed, absolutely!*

***Still?***

*No, now I am making up. By being there for him, but I feel like I have failed*.” (mother, 47 years)

As a consequence of the rollercoaster of emotions family members are going through, some also experience psychical difficulties. Because of the stressful situation, their psychical health has deteriorated. Family members genuinely feel sick or depressed when they visit their relative. Only a few actively seek consultation (e.g., by going to a psychiatrist or a psychologist) to improve their own wellbeing. Unfortunately, this has often been experienced as unhelpful because it does not change the situation of their relative and therefore does not help them as a family member to cope. Other family members found it too expensive and therefore stopped going to a psychiatrist or psychologist.

“*In 1 year, I lost 22 kg because I kept on thinking about him and the situation. […] Since 1 year and a half I am taking medication and I am going to a psychiatrist to talk about the situation. […] Several times, I have been admitted to a psychiatric hospital*.” (mother, 62 years)

Notably, differences can be noticed between mothers and fathers concerning the impact of the situation. Many mothers will take on a more advocating role, striving for the best care for their relative, while fathers are often more introverted, because they are dealing more internally with the emotions they experience. Some citations of mothers illustrate this:

“*It is my child, you know. I think it is just maternal. Unlike him [points to the father], I would say he resigns himself to it*.” (mother, 56 years)

“*Mothers are strong, especially when it concerns their son or their daughter*.” (mother, 47 years)

“***What was your way of coping with the situation?***

*You get used to it, strange but true. I also wanted to put the situation away in the long run, that I said let's keep quiet because we cannot do anything about it anyway*.” (father, 65 years)

Interestingly, family members of a person subjected to a security measure for a longer period describe how they were able to cope better with the situation. Somehow they have learned how to protect themselves against these overwhelming emotions. There is diversity between families in the coping strategies and strengths used to handle the situation they are confronted with. Some focus on their job, practice sports (e.g., fitness, cycling, running, yoga, …) or go on a (little) trip abroad. Other families actively try to alter the situation by writing e-mails and letters to policymakers, human rights organizations or (forensic) psychiatric settings. Some family members have turned to writing down their own experiences to vent their emotions and deal with what has happened. These people think about publishing or doing something with their narrations in the future.

“*I have written mails and letters, continuously I am writing, because when I am writing these things down it is a way of ventilating my emotions*.” (mother, 47 years)

“*I am afraid that it is too late for my son, I do not see it getting better. That is very difficult to accept, but at a certain point you have to be able to place it for yourself. […] That is why I now focus more on my profession, because it is a good outlet and I also go cycling a lot. […] My daughter and the fact that she is doing well, is also something that lifts me up*.” (father, 59 years)

#### Social isolation and (not) feeling supported by others

Not only do participants describe the ways in which they cope, they also discuss the stigma related to a security measure and the label of being Not Criminally Responsible. While some family members outline how they do not experience the security measure as a stigma and do not want to hide it from others, others conceal the situation they are confronted with. The latter explains this as caused by double stigma, i.e., that their relative is not only seen as “a psychiatric patient” but also as a “criminal.” It is noticeable how at the start of the security measure many family members do not seem to share their experiences with other people because they believe the situation will change soon. Therefore, they carefully select to whom they tell something, as they want to avoid the community “talking” about it. Many have the experience that they are being judged by others and that people gossip.

“*Before, we experienced more stigma. You say to your friends and family that he is on holidays, that he is sick… We tried to hide the situation. […] But still, we do not talk about the internment. Why should they know that he is interned? That is irrelevant*.” (father, 70 years)

Inherent to the feelings of stigma, social support is also discussed. Regularly, family members feel socially isolated from friends and family and do not feel supported. Often, family members only have contact with a single supportive friend or family member.

“*You isolate yourself from others, you do not want to see anybody anymore. Every day you have to wear a mask when you go to your work, because nobody knows the situation you are in. You know, I am not ashamed to tell everybody my son is in a psychiatric hospital, but that he is involved in a judicial procedure and that he is a criminal, that is difficult to speak about*.

***Do you experience the judicial procedure as an extra label?***

*Yes, yes, yes, absolutely! […] I try to disguise with psychiatry …*” (mother, 47 years)

One participant described her faith as an important source of support. In her case, she attends the Church every week to meet people.

“*I am going to the Church, because I am religious and I have got there a lot of people that support me*.” (mother, 61 years)

Furthermore, lawyers that support their relative are sometimes experienced as supportive, more particularly when they listen to family members' complaints and are able to positively change something for the PNCR.

“*My lawyer is currently doing his very best […] I get a lot of support from him. I can just call him and ask him everything I want to know and he explains it to me. […] He says we are going to do that, we are just going to try to ask for ambulatory care. If they say no, then so be it, but he says, we are going to try*.” (partner, 57 years)

#### Being not alone and sharing experiences

The participants mentioned coming into contact with other family members of PNCR as supportive and, as a result, they feel less alone. As a consequence, some family members claim that it would be helpful to see other families regularly during, for example, a discussion evening. Others already actively attend activities organized by a non-profit organization for family members of people experiencing a severe mental illness.

“*I went to the information days organized by a non-profit organization for family members and I saw other parents, normal people who also had a son or daughter who was interned. Because before you think I am not normal, I am marginal… It looks like everyone has his perfect family and only in your family it goes wrong. And you think you are alone in the world, but that is not true*.” (mother, 47 years)

Many family members want to meet people who are coping with a similar situation, but find it difficult, as only sparsely and often in geographically distant areas, activities are organized for this group of people in Belgium.

“*I went to the information days organized by the Flemish family organization. They give you information, but you also come into contact with other family members. On these days, I saw that there are other parents, normal people, with relatives who are also patients who committed an offense. Because you think, I am marginal, I am not normal … everyone else has his perfect family and in mine everything goes wrong […]*.

***So you have contact with other families in a similar situation, only by going to those*** ***information days?***

*Yes, only at those information days, but unfortunately, they are often far away and I have to drive for a long time*.” (mother, 47 years)

### Contemplating the future

When the participants in this study are asked about the(ir) future, they are not always hopeful. Many feel that their relative will not recover anymore. The majority has no or little hope left as they have been disappointed by professionals or their relative so many times in the past.

“*You dare not to hope… Because you get disappointed. It is an agony, a severe agony. Because you have nowhere a contact point. Now I am glad I can talk about it, just to say what is going wrong*.” (partner, 57 years).

Some try to live from day to day, because they are fearful to plan things in the distant future. Besides, at times family members and especially parents reflect on the question: “who is going to take care of my child in the future?.” They hope their child will continue to be supported by mental healthcare professionals, as they do not want siblings to take over caring responsibilities. Finally, family members of younger people are afraid the life of their child is “over.” They hope their relative will get new chances to get a job, yet some of them already experienced huge difficulties concerning their social and professional rehabilitation. In general, they hope for a “normal” life for their relative.

“*There is enormous difficulty for these people to reintegrate. Because it must be adapted, that they are under the effects of drugs. It is not easy. These are people who are under-trained because they have had difficulties in their schooling. They are already arriving at the bottom of the basket with no training. And here we already have difficulties to reintegrate people without mental disorders. In addition to those with mental disorders, who are reputable and spotted, they will be dismissed sooner than others if they apply*.” (father, 66 years)

## Discussion

This qualitative study investigates the experiences of family members regarding the care trajectories of their relatives subjected to a security measure and focuses on family perspectives and family recovery processes. We aimed to understand how family members currently experience their involvement in care, what role they envision for themselves in taking care of their relative, how they see their involvement in the social (re)integration process and how their experiences and reflections are related to family recovery. During the interviews the participants ventilated their emotions and frustrations regarding their involvement in the care trajectories of their relatives, which are in line with research on the experiences of family members of persons with a severe mental illness [e.g., (34, 43–45)]. It is important to remark that when reflecting on the major themes of this study, several themes are similar to recovery experiences of family members of persons with a severe mental illness in general (i.e., emotional and social burdens). Still, it is worth pointing out that these diverse themes are important issues for family members of PNCR as well.

Family members of PNCR face, in comparison to family of persons with a severe mental illness, diverse additional challenges. They are confronted with a security measure of undefined duration, causing many insecurities, powerlessness, social discrimination and isolation. In this study, as well as in other international studies, they feel double stigmatized by society and professionals because of the offense being committed (6, 14, 15). Family members mentioned the shortages within “the system” (i.e., application of security measure) in general. Evidently, there is a lack of information on the meaning and consequences of the security measure, which is also highlighted in other research studies [e.g., (18, 46)]. However, this measure is at times also perceived by family members as positive, as an opportunity for PNCR to receive much needed care. Still, many are not convinced this measure will support the recovery of their relative (7, 9). Moreover, family members want to be heard and consulted by mental healthcare professionals. Like mentioned in other research studies, family members are striving for clarity in confidentiality policies of care facilities and need more support from and engagement with mental healthcare professionals (47). Making this possible would help them in gaining information, to cope with the situation and in taking on their role in the social (re)integration of their relative. On top of those feelings and issues, they have to deal with the probation requirement of their relative and how this impacts his/her care pathway (e.g., being referred to an inpatient ward with limited visit possibilities, …) and recovery process (27, 28). In Belgium, especially since the new law (in 2014) the focus has been shifted toward taking care of the PNCR besides the protection of society. In addition, the role of family members in this reform has been more considered. In 2020 a multidisciplinary guideline has been developed to make a “family reflex” possible in (forensic) mental healthcare. Professional mental healthcare, not only in Belgium, but also in other countries, needs to shift toward integration, participation and information of family members (48, 49).

Looking at the results of this study, it is clear that family members desire better communication and collaboration with and between different care settings. They experience their relationship with mental healthcare professionals as disappointing, which is frequently described in other research studies [e.g., (9, 15, 16, 47)]. They feel frustrated, not being heard, not having a voice in the care trajectory of their relative and therefore not being accepted as a partner in care by mental healthcare professionals (35, 50). Family members in this study strive for a “shared partnership” [see (51)] between professionals and themselves, where they can be seen as equal partners, fighting for the best possible quality of life for the PNCR. Therefore, to enable PNCR to achieve inclusive citizenship it is important to involve family members as partners in care, which will also help to support the family recovery process (35). Landeweer et al. [(48), p. 2] state that “*family involvement is an activity that requires collaboration and fine-tuning between thee stakeholders: i.e., the professionals, the person with severe mental illness and the family, the so-called ‘triadic collaboration'*.” Unfortunately and compared to general healthcare, the involvement of family members in mental healthcare differs, with various barriers being mentioned (e.g., how to handle confidentiality, how to develop mutual trust and understanding, …) [(48), p. 7].

Letting family members take on an advocacy role and installing collaboration possibilities between mental healthcare professionals and family members seems in that sense necessary. Finlay-Carruthers et al. [(7), p. 1,540] described in their research that family members “*want to be treated as a resource*.” Yet, medical confidentiality is regarded as a barrier for professionals to involve family members or give them information about the care trajectory of the relative (9, 13, 17, 26, 48). Therefore, it is essential that professionals “*understand potential conflicts regarding confidentiality and the ways in which these conflicts can be resolved*” [(52), p. 235]. Some studies mention that involving and informing family members is only possible after receiving an informed consent of the PNCR (53, 54). Others, state that if the PNCR does not explicitly oppose the sharing of information between professionals and family members, a shared partnership would be possible without consent (55).

Family members in this study pointed to the fact that they need support and help themselves in order to be able to cope with the situation they are confronted with. Throughout the interviews, the participants mentioned different phases of family recovery processes they are going through in line with those described by Spaniol and Nelson (35), making family recovery an important concept to reflect upon within forensic mental healthcare. In this study we found that family members feel “changed” as a person, feel unhappy and experience diverse social and emotional burdens. This aligns the phase of shock, discovery and denial (35) as they are confronted with a rollercoaster of emotions (9) and physical difficulties caused by the situation. Many family members mentioned that coming into contact with other families who are dealing with the same situation feels supportive. They would find it beneficial if family counseling is provided in the setting were there relative is staying to those family members who need it. More in particular, to gain insight in the situation and to feel recognized by professionals. This lines up with phase two of family recovery: recognition and acceptance (35). Empowering family members, when they are going through phase two and phase three (i.e., coping) of family recovery, is being considered important for professionals. While coping with the situation, many family members still experience hope, which is seen as an important source of strength and essential to come to self-care, to gain more insight into the situation and to undertake a more proactive role in letting their voices be heard by professionals and politicians (cf. phase four of family recovery) (7, 9, 11, 35). This study indicates that a family recovery process is not linear and can be rather described as a “bumpy road.” For instance, coping mechanisms are experienced at initial stages of the subjection to a security measure, while different “shocks” prevail throughout the care trajectory of their relative.

If we compare these results to the study of De Pau et al. (29) on the perspectives of PNCR themselves, it becomes clear that both PNCR and their family members feel insufficiently informed by care professionals and judicial actors, in addition to lacking a voice in the decision-making process. Furthermore, transition moments in the forensic care trajectory seem to hamper rather than stimulate the recovery process of PNCR. While these persons regularly rely on the support of their family members, the latter encounter multiple barriers (e.g., medical confidentiality, lack shared-decision making) to realize this support. These findings definitely demonstrate the need for a procedurally just approach, not only toward PNCR but also their families. A procedurally just approach is an interaction characterized by the experience of fairness, having a voice, having your view taken into account, being treated with respect, sensing a genuine concern and receiving sufficient information about procedures (56, 57). Wittouck and Vander Beken (58) state that forensic mental health professionals and judicial actors are power holders in forensic care trajectories and thus mediate these interactions. More critically, they argue that a procedurally just approach is an essential precondition for recovery in forensic mental healthcare. Yet, considering the findings of this study, we claim that a procedurally just approach toward families of PNCR is equally necessary and serves as a precondition for family recovery.

Focusing in this study on the perspectives and experiences of family members, it ensures their voices are being heard. Although this is the strength of the study, it can also be seen as a limitation because the opinion of professionals on family perspectives and family recovery is absent. Therefore, it would be interesting for future research to pay attention to the voices of professionals. How do they look at family recovery in forensic mental healthcare, at empowering and supporting family members of PNCR and at the idea of a shared partnership? Further, this study includes family perspectives, but looking at the participants, most of them are mothers, which is similar to other research studies [e.g., (6, 9)]. Therefore, future research should pay more attention to perspectives of other family members (like fathers, siblings, partners, …) which would be an added value to the scientific research on family perspectives and experiences in forensic mental healthcare. At last, the participants in this study are family members who are fighting for the rights and best quality of life for their relative and who can be aligned in the last phase of family recovery. However, there are also other family members, still struggling with the security measure of their relative and the consequences. These family members are hard to reach, because of the social and emotional burdens they experience. Nonetheless, their voices and the fact that they are often in the first phases of family recovery, would be interesting to listen to and capture.

To conclude, this study clearly states that much more can be achieved to support families and their recovery process. Empowering families in their caring role, inviting them for a consultation, informing them throughout the care trajectory of their relative and collaboratively hearing their voices are pathways for the future. Yet, a procedural just approach can possibly challenge the medical confidentiality, but suggests that much more can be achieved in treatment when reflecting on a “shared partnership.” Initiatives like Family Support Groups (59), family psychoeducation and inclusion (60) or the “*Trialogue*” movement (22) can be seen as examples. Again, this is not only specific for forensic mental healthcare, but should also be considered in general mental healthcare [e.g., (61, 62)]. Moreover, out of the idea of “socialization of care,” it is important for both future research and practice to further reflect on how family members' voices can be heard and on how they can be supported and involved not only in mental healthcare treatment, but also in the society in general.

## Data availability statement

The raw data supporting the conclusions of this article will be made available by the authors, without undue reservation.

## Ethics statement

The studies involving human participants were reviewed and approved by Ethics Committee of Ghent University Hospital. The patients/participants provided their written informed consent to participate in this study.

## Author contributions

SR worked together with MDP on the study design, data collection, and the data analysis. SR took the lead in writing up the paper, together with MDP. FDM, PN, FVL, and WV were contacted to review the analysis of the results and to review all the different parts of the paper. All authors read the paper several times by using several internal review rounds.

## Footnotes

^1^Families = all participants belonging to one family, which means that also couples participated.

^2^The topic list is available upon request to the first author.

## References

[1] De Pau M Nicaise P Bourmorck D Vanderplasschen W Vander Laenen F. Organizing forensic mental health care delivery: putting the trinitarian model of therapeutic security to the test. Int J Forensic Ment Health. (2021) 20:291–302. 10.1080/14999013.2021.1876795

[2] Tucker IM Brown SD Kanyeredzi A McGrath L Reavey P. Living “in between” outside and inside: the forensic psychiatric unit as an impermanent assemblage. Health Place. (2018) 55:29–36. 10.1016/j.healthplace.2018.10.009 30466813

[3] Van Roeyen S Van Audenhove S Vander Laenen F. Desistance bij wetsovertreders met een psychiatrische problematiek: tussen droom en daad staat de internering in de weg. Cahiers Politiestudies. (2016) 3:171–90.

[4] Pillay SM Oliver B Butler L Kennedy H. Risk stratification and the care pathway. Ir J Psychol Med. (2008) 25:123–7. 10.1017/S0790966700011228 30282248

[5] Liégeois A Van Audenhove C. Ethische dilemma's in de vermaatschappelijking van de GGZ in Vlaanderen. Tge. (2001) 11:107–13.

[6] Askola R Louheranta O Soininen P Putkonen H Astedt-Kurki P Paavilainen E. The offense as perceived by the parents of forensic psychiatric patients. Issues Ment Health Nurs. (2017) 38:705–11. 10.1080/01612840.2017.1326993 28613093

[7] Finlay-Carruthers G Davies J Ferguson J Browne K. Taking parents seriously: the experiences of parents with a son or daughter in adult medium secure forensic mental health care. Int J Ment Health Nurs. (2018) 27:1535–45. 10.1111/inm.12455 29573117

[8] Paradis-Gagné E Holmes D Jacob JD. Caring for a violent relative with severe mental illness: a qualitative study. J Res Nurs. (2020) 25:664–76. 10.1177/1744987120937409 34394689 PMC7932458

[9] Rowaert S Vandevelde S Lemmens G Audenaert K. How family members of mentally ill offenders experience the internment measure and (forensic) psychiatric treatment in Belgium: a qualitative study. Int J Law Psychiatry. (2017) 54:76–82. 10.1016/j.ijlp.2017.05.003 28528986

[10] Wittouck C Vander Laenen F Vandevelde S Rowaert S Aga N Van Roeyen S. Lived-experiences-and-strengths-based strategies for persons with mental illness who offended and their family members. Freedom Fear. (2019) 15:48–57. 10.18356/93d07384-en

[11] Chemerynska N Arsuffi L Holdsworth E. Ascertaining the needs of carers of forensic psychiatric inpatients through their experience of navigating mental health services: guidance for service providers. J For Psychol Res Pract. (2020) 21:3. 10.1080/24732850.2020.1851546

[12] Robinson L Haskayne D Larkin M. How do carers view their relationship with forensic mental health services? J For Psychol Res Pract. (2017) 17:232–48. 10.1080/24732850.2017.132680425811867

[13] Rowaert S Vandevelde S Lemmens G Vanderplasschen W Vander Beken T Vander Laenen F. The role and experiences of family members during the rehabilitation of mentally ill offenders. Int J Rehabil Res. (2016) 39:11–9. 10.1097/MRR.0000000000000152 26756851

[14] Tsang HWH Pearson V Yuen CH. Family needs and burdens of mentally ill offenders. Int J Rehabil Res. (2002) 25:25–32. 10.1097/00004356-200203000-00004 11953712

[15] Pearson V Tsang HWH. Duty, burden, and ambivalence: families of forensic psychiatric patients in Hong Kong. Int J Law Psychiatry. (2004) 27:361–74. 10.1016/j.ijlp.2003.08.001 15271530

[16] Nordström A Kullgren G Dahlgren L. Schizophrenia and violent crime: the experience of parents. Int J Law Psychiatry. (2006) 29:57–67. 10.1016/j.ijlp.2004.07.002 16278016

[17] Ridley J McKeown M Machin K Rosengard A Little S Briggs S. Exploring Family Carer Involvement in Forensic Mental Health Services. Scotland: Support in Mind Scotland, University of Central Lancashire, and Forensic Mental Health Services Managed Care Network (2014). p. 1–87.

[18] Tingleff E B Rowaert S Vinding S Vestphal T, K.. “It's still our child”: a qualitative interview study with parent carers in forensic mental health. Arch Psychiatr Nurs. (2022) 41:12–131. 10.1016/j.apnu.2022.07.017 36428040

[19] Dewaele C De Maeyer J Reynaert D Beelen S Vandevelde S Meesen D. Vermaatschappelijking: laveren tussen kansen en bedreigingen. Geraadpleegd (2015). Available online at: https://sociaal.net/analyse-xl/vermaatschappelijking (accessed October 18, 2017).

[20] Kim HW Salyers MP. Attitudes and perceived barriers to working with families of persons with severe mental illness: mental health professionals' perspectives. Community Ment Health J. (2008) 44:337–45. 10.1007/s10597-008-9135-x 18437570

[21] Senneseth M Pollak C Urheim R Logan C Palmstierna T. Personal recovery and its challenges in forensic mental health: systematic review and thematic synthesis of the qualitative literature. BJPsych Open. (2022) 8:E17. 10.1192/bjo.2021.1068 34915963 PMC8715254

[22] Amering M Mikus M Steffen S. Recovery in Austria: mental health trialogue. Int Rev Psychiatry. (2012) 24:11–8. 10.3109/09540261.2012.655713 22385422

[23] Lakeman R. Practice standards to improve the quality of family and carer participation in adult mental health care: an overview and evaluation. Int J Ment Health Nurs. (2008) 17:44–56. 10.1111/j.1447-0349.2007.00510.x 18211403

[24] Rowaert S. Supporting family members of mentally ill offenders: a strengths-based approach (doctoral dissertation). Ghent University, Ghent, Belgium (2018).

[25] Canning AHM O'Reilly SA Wressell LRS Cannon D Walker J. A survey exploring the provision of carers' support in medium and high secure services in England and Wales. J For Psychiatry Psychol. (2009) 20:868–85. 10.1080/14789940903174154

[26] MacInnes DL Watson JP. The differences in perceived burdens between forensic and non-forensic caregivers of individuals suffering from schizophrenia. J Mental Health. (2002) 11:375–88. 10.1080/09638230020023741

[27] Aga N Vander Laenen F Vandevelde S Vermeersch E Vanderplasschen W. Recovery of offenders formerly labeled as not criminally responsible : uncovering the ambiguity from first-person narratives. Int J Offender Ther Comp Criminol. (2019) 63:919–39. 10.1177/0306624X17730617 28893122

[28] Aga N Rowaert S Vander Laenen F Vandevelde S Vander Beken T Audenaert K. Connectedness in recovery narratives of persons labeled not criminally responsible : a qualitative study. Int J Forensic Ment Health. (2021) 20:303–16. 10.1080/14999013.2021.1880503

[29] De Pau M Mertens A Bourmorck D Vanderplasschen W Nicaise P Vander Laenen F. Crushed by the Belgian system: lived experiences of forensic care trajectories by persons labelled as not criminally responsible. Int J Law Psychiatry. (2020) 68:101539. 10.1016/j.ijlp.2019.101539 32033703

[30] Nicaise P Bourmorck D De Pau M Vander Laenen F Vanderplasschen W Lorant V. Are mentally disordered offenders admitted to appropriate secure settings according to security needs? Across sectional study in Belgium. Int J Forensic Ment Health. (2022) 21:307–19. 10.1080/14999013.2021.2007430

[31] Bradshaw S Shumway ST Wang EW Harris KS Smith DB Austin-Robillard H. Hope, readiness, and coping in family recovery from addiction. J Groups Addict Recov. (2015) 10:313–36. 10.1080/1556035X.2015.1099125

[32] Dekkers A De Ruysscher C Vanderplasschen W. Perspectives on addiction recovery: focus groups with individuals in recovery and family members. Addict Res Theory. (2020) 28:526–36. 10.1080/16066359.2020.1714037

[33] Hayes ND Bradshaw SD Mullet N Smith JA Shumway SS. Exploring family member influence on change in addiction treatment, a dyadic analysis. Alcohol Treat Q. (2019) 37:377–95. 10.1080/07347324.2018.1534534

[34] Spaniol L. The pain and the possibility: the family recovery process. Community Ment Health J. (2010) 46:482–5. 10.1007/s10597-010-9315-3 20464490

[35] Spaniol L Nelson A. Family recovery. Community Ment Health J. (2015) 51:761–7. 10.1007/s10597-015-9880-6 25947133

[36] Ting To W Vanheule S De Smet S Vandevelde S. The treatment perspectives of mentally ill offenders in medium- and high-secure forensic settings in Flanders. Int J Offender Ther Comp Criminol. (2015) 59:1605–22. 10.1177/0306624X14566355 25583981

[37] Völlm BA Clarke M Herrando VT Seppänen A Gosek P Heitzman J. European Psychiatric Association (EPA) guidance on forensic psychiatry: evidence based assessment and treatment of mentally disordered offenders. Eur Psychiatry. (2018) 51:58–73. 10.1016/j.eurpsy.2017.12.007 29571072

[38] Heimans H Vander Beken T. De nieuwe interneringswet van 5 mei 2014. In:Casselman J De Rycke R Heimans H, editors. Internering: Nieuwe Interneringswet en Organisatie van de Zorg. Bruges: Die Keure (2015). p. 49–110.

[39] Pesout K Pham T. Forensic psychiatric care in Belgium. In:Völlm B Braun P, editors. Long-Term Forensic Psychiatric Care: Clinical, Ethical and Legal Challenges. Cham: Springer (2019). p. 261–72. 10.1007/978-3-030-12594-3_18

[40] Vanlinthout E Coppens E Opgenhaffen T Scheveneels S Put J Van Audenhove C. Een multidischiplinaire richtlijn om naasten sterker te betrekken in de geestelijke gezondheidszorg. (2020). Available online at: https://cdn.nimbu.io/s/5s8z9pq/channelentries/sm2apk4/files/Multidisciplinaire%20richtlijn_betrekken%20naasten.pdf?lw3ci0o. Steunpunt Welzijn, Volksgezondheid en Gezin. Publicatienr. 2020/15b (accessed November 21, 2022).

[41] Braun V Clarke V. Using thematic analysis in psychology. Qual Res Psychol. (2006) 3:77–101.

[42] Boyatzis R. Transforming Qualitative Information: Thematic Analysis and Code Development. Thousand Oaks, CA: Sage (1998).

[43] Corsentino EA Molinari V Gum AM Roscoe LA Mills WL. Family caregivers' future planning for younger and older adults with serious mental illness (SMI). J Appl Gerontol. (2008) 27:466–85. 10.1177/0733464808315290

[44] van der Sanden RLM Pryor JB Stutterheim SE Kok G Bos AER. Stigma by association and family burden among family members of people with mental illness: the mediating role of coping. Soc Psychiatry Psychiatr Epidemiol. (2016) 51:1233–45. 10.1007/s00127-016-1256-x 27357819 PMC5025495

[45] Wynaden D. The caregiving experience: how much do health professionals understand? Collegian. (2007) 13:6–10. 10.1016/S1322-7696(08)60526-0 17036449

[46] Absalom-Hornby VL Gooding P Tarrier N. Coping with schizophrenia in forensic services: the needs of relatives. J Nervous Mental Dis. (2011) 199:398–402. 10.1097/NMD.0b013e31821cd394 21629019

[47] Sampson S Foster S Majid S Völlm B. Carers of long-stay patients' perspectives of secure forensic care: an exploratory qualitative study. Int J Forensic Ment Health. (2019) 18:305–15. 10.1080/14999013.2018.1552635

[48] Landeweer E Molewijk B Hem MH Pedersen R. Worlds apart? A scoping review addressing different stakeholder perspectives on barriers to family involvement in the care for persons with severe mental illness. BMC Health Serv Res. (2017) 17:349. 10.1186/s12913-017-2213-4 28506296 PMC5433083

[49] Machin K Musgrave S Persaud K Ridley J. Carers and forensic services. Towards carers' peer support. In:Tomlin J Völlm B, editors. Diversity and Marginalisation in Forensic Mental Health Care. New York, NY: Routledge (2022). 10.4324/9781003184768-23

[50] Bradley E Green D. Invlved, inputting or informing: “Shared” decision making in adult mental health care. Health Expect. (2017) 21:192–200. 10.1111/hex.12601 28779520 PMC5750775

[51] Quah SR. Partnership: the missing link in the process of de-instituationalization of mental health care. Int J Health Serv. (2017) 47:532–49. 10.1177/0020731415614512 26588941

[52] Marsh DT Johnson DL. The family experience of mental illness: implications for intervention. Profess Psychol Res Pract. (1997) 28:229–37. 10.1037/0735-7028.28.3.22920950302

[53] Kivits P Thémines J Rohmer G. Improving patient outcome in involuntary psychiatric hospitalizations: contribution of systemic approaches. Ann Médico Psychol Revue Psychiatr. (2018) 176:489–94. 10.1016/j.amp.2017.02.016

[54] Lavoie J. Relative invisibility: an integrative review of carers' lived experiences of a family member's emergency mental health crisis. Soc Work Ment Health. (2018) 16:601–26. 10.1080/15332985.2018.1467845

[55] Fiorillo A De Rosa C Del Vecchio V Jurjanz L Schnall K Onchev G. How to improve clinical practice on involuntary hospital admissions of psychiatric patients: suggestions from the EUNOMIA study. Eur Psychiatry. (2011) 26:201–2077. 10.1016/j.eurpsy.2010.01.013 20965119

[56] Lind EA Tyler TR. The Social Psychology of Procedural Justice. New York, NY: Plenum Press (1988). 10.1007/978-1-4899-2115-4

[57] Lind EA Kanfer R Early PC. Voice, control, and procedural justice – instrumental and noninstrumental concerns in fairness judgements. J Pers Soc Psychol. (1990) 59:952–9. 10.1037/0022-3514.59.5.952

[58] Wittouck C Vander Beken T. Recovery, desistance and the role of procedural justice in working alliances with mentally ill offenders: a critical review. Addict Res Theory. (2019) 27:26–8. 10.1080/16066359.2018.1518434

[59] Rowaert S Vandevelde S Audenaert K Lemmens G. Family support groups for family members of mentally ill offenders: a pilot study. J Forensic Psychiatr Psychol. (2018) 29:762–3. 10.1080/14789949.2018.1508482

[60] Nagi C Davies J. Bridging the gap: brief family psychoeducation in forensic mental health. J Forensic Psychol Pract. (2015) 15:171–83. 10.1080/15228932.2015.1013786

[61] Fadden G. Training and disseminating family interventions for schizophrenia: developing family intervention skills with multi-disciplinary groups. Assoc Family Ther Syst Pract. (2006) 28:23–38. 10.1111/j.1467-6427.2006.00335.x

[62] McFarlane WR. Family interventions for schizophrenia and the psychoses: a review. Fam Process. (2016) 55:460–82. 10.1111/famp.12235 27411376

